# Correction: Khalaj-Hedayati et al. Identification and In Silico Characterization of a Conserved Peptide on Influenza Hemagglutinin Protein: A New Potential Antigen for Universal Influenza Vaccine Development. *Nanomaterials* 2023, *13*, 2796

**DOI:** 10.3390/nano16191272

**Published:** 2026-10-08

**Authors:** Atin Khalaj-Hedayati, Caroline Lin Lin Chua, Peter Smooker, Seyedehmaryam Moosavi, Otilia Manta, Mohamed H. Helal, Mohamed M. Ibrahim, Zeinhom M. El-Bahy, Ganden Supriyanto, Khai Wooi Lee

**Affiliations:** 1 School of Biosciences, Faculty of Health and Medical Sciences, Taylor’s University, Subang Jaya 47500, Malaysia; linlin.chua@taylors.edu.my; 2 Department of Chemistry, Faculty of Science and Technology, Airlangga University, Mulyorejo, Surabaya 60115, Indonesia; 3 Centre for Active Living, Taylor’s University, Subang Jaya 47500, Malaysia; 4 Department of Biosciences and Food Technology, School of Science, RMIT University, Bundoora 3000, Australia; peter.smooker@rmit.edu.au; 5 Department of Nanotechnology Engineering, Faculty of Advance Technology and Multidiscipline, Airlangga University, Mulyorejo, Surabaya 60115, Indonesia; m.moosavi1987@gmail.com; 6 Romanian Academy, Victor Slavescu Centre for Financial and Monetary Research, 050731 Bucharest, Romania; otilia.manta@rgic.ro; 7 Romanian Academy, CE-MONT Mountain Economy Center, 725700 Vatra Dornei, Romania; 8 Research Department, Romanian American University, 012101 Bucharest, Romania; 9 Department of Chemistry, Faculty of Arts and Science, Northern Border University, Rafha 76413, Saudi Arabia; mohammed.hlal@nbu.edu.sa; 10 Department of Chemistry, College of Science, Taif University, P.O. Box 11099, Taif 21944, Saudi Arabia; ibrahim@tu.edu.sa; 11 Department of Chemistry, Faculty of Science, Al-Azhar University, Nasr City 11884, Egypt; zeinelbahy@azhar.edu.eg

In the original publication [1], Caroline Lin Lin Chua, Peter Smooker, and Khai Wooi Lee were not included as authors. The corrected author names, Author Contributions and Funding appear here.

Atin Khalaj-Hedayati, Caroline Lin Lin Chua, Peter Smooker, Seyedehmaryam Moosavi, Otilia Manta, Mohamed H. Helal, Mohamed M. Ibrahim, Zeinhom M. El-Bahy, Ganden Supriyanto and Khai Wooi Lee.

The corrected Author Contributions statement and Funding appears here:

**Author Contributions:** Conceptualization, A.K.-H.; methodology, A.K.-H.; formal analysis, A.K.-H.; writing—original draft preparation, A.K.-H.; writing—review and editing, A.K.-H., S.M., G.S., O.M., Z.M.E.-B., C.L.L.C., P.S. and K.W.L.; supervision, G.S., C.L.L.C., P.S. and K.W.L.; project administration, A.K.-H.; funding acquisition, O.M., M.H.H., M.M.I., Z.M.E.-B., C.L.L.C., P.S. and K.W.L. All authors have read and agreed to the published version of the manuscript.
**Funding:** This research was funded by Taylor’s Internal Research Grant Scheme—Major Funding Scheme (TIRGS-MFS), Grant No. TRGS/MFS/2/2013/SBS/001; the Taylor’s Internal Research Grant Scheme—Emerging Research Funding Scheme (TIRGS-ERFS), Grant No. TRGS/ERFS/1/2018/SBS/039, from Taylor’s University, Malaysia; and the Deanship of Scientific Research, Northern Border University, Arar, Saudi Arabia, Grant No. NBU-FFR-2023-0128. Atin K-H was supported by the Taylor’s University Vice Chancellor Scholarship Award during her PhD candidature.

The authors state that the scientific conclusions are unaffected. This correction was approved by the Academic Editor. The original publication has also been updated.
